# Three Decades of Trends in Risk Factors Attributed to Disease Burden in Saudi Arabia: Findings from the Global Burden of Disease Study 2021

**DOI:** 10.3390/healthcare13141717

**Published:** 2025-07-17

**Authors:** Amal Zaidan

**Affiliations:** 1 College of Public Health and Health Informatics (CPHHI), King Saud bin Abdulaziz University for Health Sciences, Riyadh 11481, Saudi Arabia; zaidanam@ksau-hs.edu.sa; 2 King Abdullah International Medical Research Center, Riyadh 11481, Saudi Arabia

**Keywords:** risk factors, DALYs, gender variation, disease burden

## Abstract

**Objective:** This study aimed to explore the burden attributable to different groups of risk factors (environmental/occupational, behavioral, and metabolic) in Saudi Arabia that were stratified by gender and year and measured by summary exposure values (SEVs) and disability-adjusted life years (DALYs) per 100,000. **Design:** This study was structured as a systematic analysis. Methods: Using the GBD 2021 data, we extracted information on different risk factors attributed to the disease burden in Saudi Arabia to quantify the differences in exposure value (SEV) and disability-adjusted life year (DALY) rates (per 100,000) between females and males across different years. **Results:** Over the years, sustained progress in reducing the number of DALYs attributable to specific environmental and occupational risks has been observed, as well as a slight decrease in some behavioral risks. The highest disease burden was attributed to metabolic and behavioral risk factors, with body mass index being the leading risk factor for both genders. Between 1990 and 2021, the age-standardized DALY rate in those with high body mass indices increased by 168.4% and reached 3436.23 (95% UI 1878.7–5031.5) in males and increased by 125.2% to reach 2952.6 (95% UI 1456.9–4.407) in females. The age-standardized SEVs were the highest in females with a high body mass index, reaching an SEV of 57.98 (95% UI: 64.1–49.2), and in males, an SEV of 50.75 (95% UI: 57.1–42.3) was achieved. Regarding their attributable deaths in 2021, metabolic risk factors were identified as the primary contributors to NCD mortality in 2021. **Conclusions:** These results reveal persistent health disparities between males and females, underscoring the urgent need for gender-specific research, policies, and interventions. Strategies aimed at promoting health and reducing disease burden should acknowledge the unique health challenges encountered by males and females.

## 1. Introduction

Protecting lives and preventing disease, injury, and premature death involves modifying environmental, occupational, behavioral, and metabolic risk factors that contribute to their occurrence [1,2]. However, quantifying the link between an identified risk factor and its related disease outcome is complex, but it is beneficial in evaluating resource allocation decisions and developing effective strategies for these modifiable risk factors, which are crucial for enhancing public health and primary prevention [1,3].

Recently, the global estimate for the highest risk-attributable burden in terms of disability-adjusted life years (DALYs) per 100,000 was for behavioral risks, with 763 million (95% UI 650–865) DALYs, followed by metabolic risks, with 476 million (95% UI 412–541) DALYs, and 416 million (95% UI 364–469) DALYs were attributable to environmental and occupational risks [2,4], a burden that remains a significant global public health challenge in an ever-changing global health landscape. However, the most effective risk-reduction strategies are tailored to specific characteristics, including but not limited to education, sex, location, and age [2,5]. For example, it is well known that sex interacts differently with several factors and determinants, such as social, economic, and commercial determinants [6,7]. Globally, females tend to live longer, with a life expectancy difference of 5 years compared to males [8,9], but they have higher morbidity and spend a higher proportion of their total life expectancy in poor health [10,11,12]. From a risk assessment, men tend to engage in potentially detrimental behaviors, such as drug abuse and smoking, which have been linked to the role of sex hormones in social behaviors and psychological tendencies. On the other hand, females tend to be exposed to various stressors due to systemic reasons, such as a lack of societal empowerment and low social status, making them more susceptible to developing non-communicable diseases [13]. Furthermore, research has indicated that gender affects both access to healthcare and the quality of care provided by health services [14,15].

With the rapid modernization and influx of new lifestyle habits, the significant shift over the last 50 years has resulted in a progressive increase in the prevalence of obesity and non-communicable diseases in Saudi Arabia [16,17]. The economic, social, and cultural development achievements have been accompanied by lifestyle modifications, with a greater tendency towards sedentary behavior and physical inactivity, particularly among women [18,19]. Furthermore, gender has been recognized as a contributor to disparities in disease burden [20,21,22]. Women have a higher life expectancy rate of 77.4 years, which has increased compared to 75.7 years in 2021 [23]. However, the healthy life expectancy at birth (HALE) was higher for males, with averages of around 65.8 years and 65.1 years [23]. Despite the non-significant difference in HALE between the two genders, the numbers show that the HALE is more than a decade lower than the average lifespan for both genders. Furthermore, the age-standardized DALYs per 100,000 for all risk factors in 2021 for males reached 14,831.9 (95% UI 17,001.3–12,938.7), while it was 12,643 (95% UI 15,109.2–10,344.9) for females [4]. These gender variations underscore the need for gender-specific interventions. In 2016, as part of Vision 2030, the Ministry of Health initiated a health transformation of the healthcare system, with one of its main objectives being to promote health risk prevention [24]. This study used GBD data to explore the burden attributable to different risk factors (environmental/occupational, behavioral, and metabolic) that were stratified by gender and year and measured by the summary exposure value (SEV), DALY, and mortality percentage. A previous GBD study provided a country-specific report for Saudi Arabia up to 2017, covering both genders without stratification [16,17]. In this study, the differences in health between females and males will be explored. Recognizing differences in risk exposure and health outcomes by gender is essential for pinpointing interventions that can effectively reduce health inequities. This data will be interpreted and used to assess improvements, examine challenges, and provide opportunities to address gender-specific health disparities, as its valuable insights will aid in prioritizing interventions.

## 2. Methods

This study used publicly accessible data from the World Health Organization (WHO) and the Institute for Health Metrics and Evaluation’s GBD repository [25]. The Global Burden of Disease (GBD) study periodically publishes updated data that provides a comprehensive estimate and metrics of mortality and morbidity trends for 371 diseases and injuries from 1990 to 2021 in 204 countries and territories, as well as for 23 age groups from birth to age 95 years and older [4]. The GBD study complies with the Guidelines for Accurate and Transparent Health Estimates Reporting (GATHER) and is registered and approved by the University of Washington’s Institutional Review Board (study number 9060) [26]. This study extracted data on different risk factors attributed to disease burden in Saudi Arabia to quantify differences in DALY rates (per 100,000) and mortality percentages between females and males across different years. All citations and metadata used in this study are available in the (GBD 2021) Sources Tool.

### 2.1. GBD Hierarchy

The cause hierarchy in the GBD 2021 data is classified into four levels, ranging from the broadest groupings at Level 1 to the most detailed at Level 4 [4,25,27]. Similarly, the hierarchy of risk factors is classified into environmental and occupational, behavioral, and metabolic risks. These factors are also disaggregated into additional risk categories as we progress with the level of disaggregation [4,25,27,28].

### 2.2. Summary Exposure Value (SEV)

The SEV represents the age-specific risk-weighted prevalence of exposure, which was reported as age-standardized rates on a 0–100 scale for the total population, where zero means no excess risk and 100 means everyone in the population is exposed to the maximum risk. This value accounts for the severity and the proportion of the exposed population and is comparable across different risk factors with varying exposure patterns [29]:

$${SEV}_{rc}=\frac{\int_{x=1}^{u}P\left(x\right)\;RR\;\left(x\right)\;dx-1}{{RR}_{Max}-1}$$

*P*(*x*) is the density of exposure at level *x*, *RR*(*x*) is level *x* of the relative risk of exposure, and *RR_max_* is the relative risk at the 99th percentile of the global distribution of exposure [29]. The rate of change from 1990 to 2021 is calculated by comparing the estimates and then computing the results.

### 2.3. Disability-Adjusted Life Years

DALYs estimate the total health loss due to fatal and non-fatal disease burdens, enabling comparisons across diseases and injuries [30]. Each DALY represents the population’s loss of 1 year of healthy life and is calculated as the sum of YLLs (Years of Life Lost) and YLDs (Years Lived with Disability) [31,32] as follows:

DALYs = YLLs + YLDs

YLLs are the number of deaths per 100,000 people multiplied by a global standard life expectancy at the age of death, while YLDs are estimated as the combined prevalence and duration of a disease or injury weighted by a measure of disease severity (referred to as disability weights) that ranges from 0 (full health) to 1 (fatal severity) [33]. For this study, the YLLs in Saudi Arabia were calculated by multiplying the number of deaths caused by disease by the remaining life expectancy derived from the GBD standard life table [34]. Similarly, YLDs were determined by multiplying the disease prevalence by the disability weight of the person with the disability. The age-standardized rate was used to adjust for differences in the population’s age distribution for more representative outcomes. Following the same-sex disaggregation in the GBD study, which adheres to a binary framework (female or male), sex was stratified into male and female to compare trends in disease burden, and 95% uncertainty intervals (UIs) were reported for all estimates.

### 2.4. Analysis

The analysis is based on Level 1 causes and Level 2 risk factors (Table 1), as these constitute our primary outcomes of interest for which comparisons are feasible. Level 1’s broadest group presents a more generalized estimate of the attributable risk for both genders for the different causes. The prevalence of exposure, which is age-specific and weighted by risk, was provided for Level 2 risk factors, describing differences in SEVs between females and males. Following this, comparisons were made between the two genders for the most significant disease burden—DALY rate per 100,000 for the most recent year and 1990. Finally, a comparison of the attributable mortality rates for females and males in 2021 was presented for different risk factors (Level 2 of the risk factor hierarchy). All values are presented with 95% uncertainty intervals (UIs), reflecting the uncertainty surrounding the estimates presented [35]. In the GBD study, every estimate was calculated 1000 times; these data are ordered from smallest to largest, and the 25th and 975th values of the ordered data determine the 95th uncertainty level.

The data from the 2021 Global Burden of Disease (GBD) study were accessed for research purposes on 11 October 2024.

### 2.5. Ethical Approval

The Institutional Review Board at King Abdullah International Medical Research Centre (KAIMRC), Saudi Arabia, has waived this study from IRB review, due to not including human subjects or relevant data.

## 3. Results

All estimates are accessible for visual exploration through the online visualization tool GBD Compare.

### 3.1. Summary Exposure Values (SEVs)

Table 1 presents Level 2 risk factors and their risk exposure, as measured by age-standardized SEVs. In Saudi Arabia, the highest SEV in environmental and occupational risks in 1990 and 2021 was for non-optimal temperature for both genders (Table 1). The highest increase of 0.56% (0.9 to 0.26) in the rate of change was observed for females with occupational risks, and the highest increase of almost 0.09% (0.32 to −0.08) was observed for males exposed to air pollution.

In terms of metabolic risk, in 1990, the risk exposure was the highest for high LDL cholesterol in males (SEV 38.8 [95% UI 55.25 to 25.4]) and females (SEV 36.2 [95% UI 51.7 to 23.6]). In 2021, the highest SEV was observed in females with a high body mass index, reaching an SEV of 57.98 (95% UI: 64.1 to 49.2), while in males, the SEV was 50.75 (95% UI: 57.1 to 42.3). However, the rate of change between 1990 and 2021 increased substantially in females for high fasting plasma glucose, at 1.15% (1.6 to 0.72), and in males, it increased for high body mass index at 1.18% (1.4 to 0.95). However, the change in both was statistically non-significant.

Among behavioral risk factors, the SEV for low physical activity was the highest for females, at 46.3 (95% UI: 54.9 to 38.24), which has increased since 1990, with a rate of 0.31% (0.61 to 0.06). In males, the SEV for tobacco was the highest in both years (Table S1).

**Table 1:** Age-standardized SEVs in 1990 and 2021 and the rate of change over 1990–2021 for GBD Level 2 risk factors.

All Risk Factors	Female			Male		
	SEV 1990	SEV 2021	Rate of Change from 1990 to 2021 (%)	SEV 1990	SEV 2021	Rate of Change from 1990 to 2021 (%)
* Environmental and occupational risks						
δ Unsafe water, sanitation, and handwashing	23.03 (32 to 12.87)	5.5 (8.5 to 2.6)	−0.75 (−0.65 to −0.8)	23 (3 to 12.9)	5.5 (8.5 to 2.6)	−0.75 (−0.65 to −0.83)
δ Occupational risks	0.45 (0.65 to −0.37)	0.76 (0.97 to 0.62)	0.56 (0.9 to 0.26)	3.98 (4.56 to 3.4)	3.8 (4.4 to 3.3)	−0.034 (0.035 to −0.1)
δ Air pollution	37.79 (46.8 to −29.2)	41.2 (49.3 to 33.7)	0.09 (0.31 to −0.08)	39.29 (48.3 to 30.45)	42.9 (51.2 to 35.2)	0.093 (0.32 to −0.08)
δ Other environmental risks	35.87 (45.32 to 2.88)	23.3 (29.7 to 2.5)	−035 (0 to −0.54)	52.12 (62.9 to 2.88)	32.1 (39.5 to 2.5)	−0.4 (0 to −0.448)
δ Non-optimal temperature	79.62 (87.4 to 70.78)	86.5 (94.9 to 74.7)	0.086 (0.14 to 0.018)	79.6 (87.4 to 70.8)	86.5 (94.9 to −74.7)	0.086 (0.14 to 0.018)
* Metabolic risks						
δ Kidney dysfunction	3.03 (3.95 to 2.39)	3 (3.9 to 2.45)	0.008 (0.07 to −0.04)	2.95 (3.9 to 2.3)	2.9 (3.9 to 2.4)	−0.0016 (0.05 to −0.05)
δ High LDL cholesterol	36.2 (51.7 to 23.6)	46.9 (65.7 to 31.9)	0.29 (0.38 to 0.23)	38.8 (55.25 to 25.4)	47.8 (66.7 to 32.1)	0.23 (0.31 to 0.17)
δ High fasting plasma glucose	14.1 (17.2 to 10.32)	30.3 (37.1 to 21.8)	1.15 (1.6 to 0.72)	17.25 (21.08 to 13.3)	29.7 (35.8 to 22.5)	0.72 (1.17 to 0.34)
δ High systolic blood pressure	33.82 (49.9 to 20.8)	22.2 (33.5 to 14)	−0.34 (−0.03 to −0.56)	36 (51.23 to 24.1)	35.7 (49 to 24.6)	−0.01 (0.2 to −0.24)
δ High body mass index	29.7 (33.8 to 25.32)	57.98 (64.1 to 49.2)	0.95 (1.12 to 0.78)	23.27 (27.1 to 20.1)	50.75 (57.1 to 42.3)	1.18 (1.4 to 0.95)
δ Low bone mineral density	27.14 (35.1 to 20.14)	23.58 (30.8 to 17.1)	−0.13 (0.05 to −0.3)	20.8 (28.3 to 14.5)	17.9 (25.1 to 11.6)	−0.13 (0.08 to −0.32)
* Behavioral risks						
δ Drug use	0.49 (0.76 to 0.23)	0.5 (0.76 to 0.22)	0.037 (0.2 to −0.1)	0.35 (0.48 to 0.22)	0.33 (0.44 to 0.21)	−0.05 (0.05 to −0.15)
δ Child and maternal malnutrition	12.73 (18.2 to 8.27)	11.9 (16.7 to 7.9)	−0.06 (0.05 to −0.17)	7.36 (12.9 to 3.9)	3.3 (5.8 to 1.8)	−0.55 (−0.5 to −0.6)
δ Childhood sexual abuse and bullying	4.97 (7.3 to 3.5)	6.5 (10.45 to 4.1)	0.30 (0.58 to 0.067)	7.15 (12.47 to 3.8)	8.8 (16.2 to 4.26)	0.23 (0.54 to −0.2)
δ Tobacco	18.9 (21.15 to 16.7)	21 (23.5 to 18.7)	0.11 (0.28 to −0.4)	32.42 (36.3 to 28.76)	32.78 (36.36 to 29.2)	0.01 (0.15 to −0.1)
δ High alcohol use	0.5 (1.49 to 0.16)	0.41 (1.28 to 0.13)	−0.18 (−0.02 to −0.43)	1.37 (4.3 to 0.51)	0.99 (3.29 to 0.34)	−0.27 (−0.12 to −0.5)
δ Dietary risks	27.64 (36.4 to 19.1)	31.3 (40.6 to 22.8)	0.13 (0.26 to 0.04)	28.65 (39.37 to 19.6)	31.7 (42.6 to 22.1)	0.1 (0.2 to 0.03)
δ Low physical activity	35.3 (42.5 to 28.8)	46.3 (54.9 to 38.24)	0.31 (0.61 to 0.06)	20.8 (26.6 to 15.5)	26.9 (34.3 to 20.5)	0.3 (0.74 to −0.03)
δ Intimate partner violence	24 (31.7 to 13.66)	23.9 (31.3 to 13.6)	−0.002 (0.7 to −0.3)			

* Level one risk factors and δ are Level two risk factors from the GBDs.

### 3.2. Ranking of Leading Risk Factors for Females and Males Between 1990 and 2021

**Figure 1:**
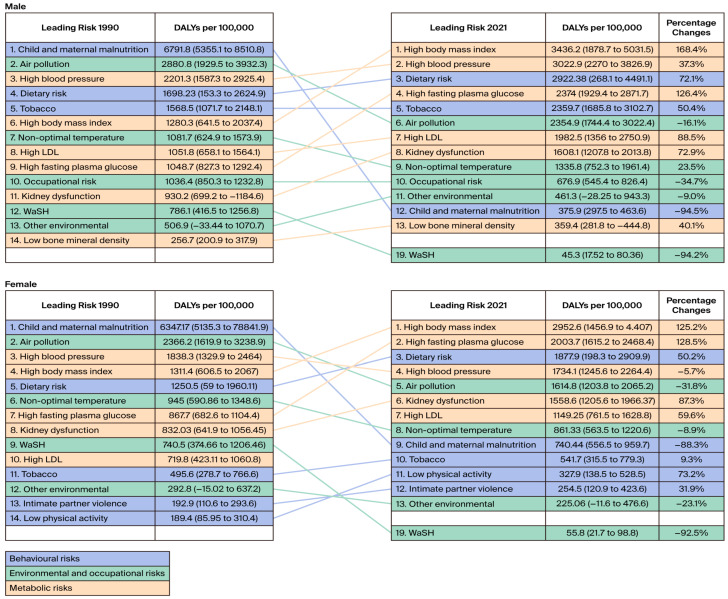
Leading risk factors by attributable DALYs (per 100,000) for all ages and percent change in DALYs from 1990 to 2021 in Saudi Arabia.

The pattern regarding DALY burden attributed to risk factors varied considerably by gender and over time (Figure 1). Further disaggregation to Level 2 of the risk-attributable burden estimate revealed that metabolic risks were the dominant leading risk factors among both genders in 2021 (Figure S1). Between 1990 and 2021, the DALY rate for those with high body mass indices increased by 168.4% and reached 3436.23 (95% UI 1878.7 to 5031.5) in males, while it increased by 125.2% to reach 2952.6 (95% UI 1456.9 to 4.407) in females. Other metabolic risk factors included high blood pressure, fasting plasma glucose, low-density lipoprotein (LDL), and kidney dysfunction (Figure 1). For behavioral risk factors, dietary risk ranked third in both genders, and the DALY rate increased over the years in both groups. The ranking of tobacco remained unchanged for males over the years, ranking fifth in both 1990 and 2021. However, the number of DALYs (per 100,000) increased from 1568.5 (95% UI: 1071.7 to 2148.1) to 2359.7 (95% UI: 1685.8 to 3102.7). In females, tobacco ranked tenth, with a minimum increment change over the years. Other behavioral risk factors, including low physical activity and intimate partner violence, which were among the leading risk factors in females, showed a positive increase in the DALY rate over the years, with values of 73.2% and 31.9%, respectively. Among the environmental/occupational risk factors, air pollution attributed to DALYs decreased in both groups, and occupational risk was present in males, leading to a risk rate of 676.9 DALYs per 100,000 (95% UI 545.4 to 826.4). 

### 3.3. Attributable Burden in 2021 for Different Risk Factors

**Figure 2:**
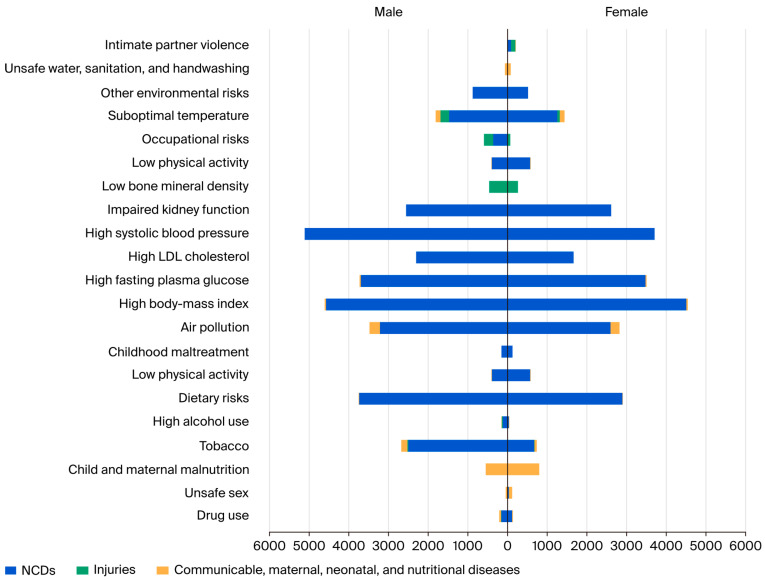
Age-standardized DALYs per 100,000 for Level 2 risk factors by cause in males and females in Saudi Arabia, 2021.

In terms of Level 2 risk factors, Figure 2 (Figure S2) compares the age-standardized DALYs per 100,000 for major conditions in 2021. Metabolic risk factors such as high systolic blood pressure in males were the highest with 5113.2 (95% UI 6099.6 to 4103.8) DALYs, and high body mass index was the highest risk factor for females with 4509.36 (95% UI 6747.9 to 2301.5) DALYs per 100,000, respectively; both were attributed to the disease burden of non-communicable diseases (NCDs). Intimate partner violence is a risk factor that was only observed among females and was associated with an injury burden of 105.6 (95% UI 151.2 to 68.8), an NCD burden of 92.7 (95% UI 217.3 to 0.3), and a communicable, maternal, neonatal, and nutritional disease burden of 8.2 (95% UI 12.9 to 4.1) DALYs per 100,000, respectively. However, most risk factors with higher DALY rates were for males, except for four behavioral risk factors, namely low physical activity in NCDs, at 567.5 (95% UI 895.6 to 229.1) and the burden of communicable, maternal, neonatal, and nutritional diseases, at 3.3 (95% UI 6.7–1); child and maternal malnutrition at 796.2 (95% UI 1007.3 to 610.2); intimate partner violence with an injury burden of 105.6 (95% UI 151.2–68.8); and a communicable, maternal, neonatal, and nutritional disease burden of 8.2 (95% UI 12.9 to 4), while unsafe sex reached 86.3 (95% UI 98.9 to 78.4) DALYs per 100,000. The metabolic risk factors that have a higher DALY (per 100,000) value for females were impaired kidney function, at 2612.1 (95% UI 3241.3 to 2063), high BMI, at 38.4 (95%UI 80.2 to 10.5), and dietary risk, at 5.86 (95% UI 11.3 to 1.7). Finally, unsafe water, sanitation, and handwashing from the environmental risk factors showed a higher DALY (per 100,000) value for females, at 76.7 (95% UI 136.4 to 29.9).

### 3.4. Attributable Deaths in 2021 for Different Risk Factors

**Figure 3:**
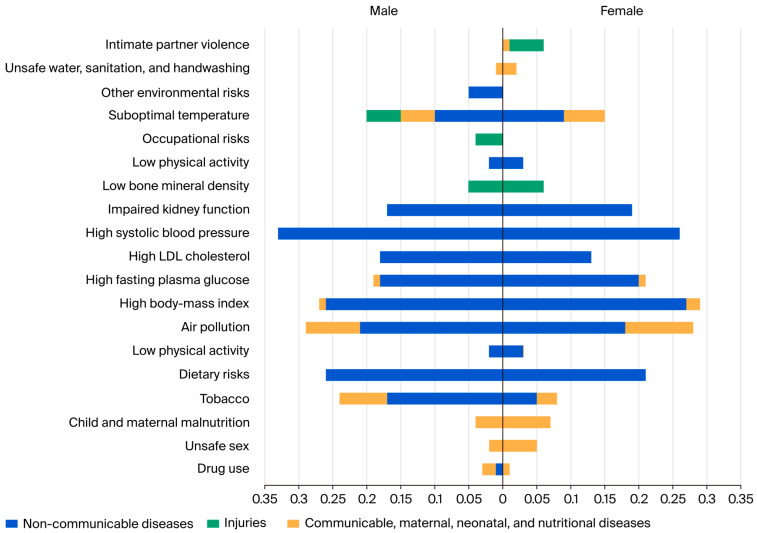
Percentage of deaths attributable to Level 2 risk factors by cause and gender in Saudi Arabia in 2021.

Level 2 risk factors contributing to the death percentage are illustrated in Figure 3 (Figure S3). In 2021, the leading risks attributable to deaths in females were high body mass index, at 26.7% (36.2 to 15.4), high systolic blood pressure, at 25.5% (31.4 to 20.4), and dietary risk, at 21.1% (31.5 to 2.5). For males, the leading risks attributable to deaths in 2021 were high systolic blood pressure, at 33.4% (38.8 to 27.4), high body mass index, at 26.2% (35.9 to 15.3), and dietary risk, at 26.14% (37.8 to 2.6). For both genders, NCDs have the most significant burden, and the leading risks attributable to death in 2021 were metabolic risk factors.

## 4. Discussion

Drawing upon the GBD 2021 study, the risk-attributable burden expressed in DALYs per 100,000 was identified for the 1990–2021 period for both genders, in which males had higher risk-attributable burdens, except in some cases. Over the years, sustained progress in reducing the number of DALYs attributable to specific environmental and occupational risks has been observed, as well as a slight decrease in some behavioral risks. However, the number of DALYs and the percentage of mortality attributable to metabolic risks increased over the same period, reflecting changes in demographics and lifestyle, which was also reflected in the increase in the rate of change in SEVs between 1990 and 2021. Although most were non-statistically significant, the association between metabolic risk and disease burden was clinically relevant. For example, the rate of change for high fasting plasma glucose in females between 1990 and 2021 was 1.15% (1.6 to 0.72). Still, the clinically relevant finding indicates that the incidence of high fasting plasma glucose in females has increased over the years [36]. Similarly, the rate of change for high body mass index for males was 1.18% (1.4 to 0.95), but the literature findings indicate an increased incidence over time [37]. However, the leading risks in 2021 were metabolic risk factors in both groups, with males facing a higher attributable risk burden. Further, most of these risk factors are modifiable and attributed to non-communicable diseases, a finding that aligns with previous GBD studies that focused on Saudi Arabia in 2014 and 2020 [16,17]. However, the findings of this study suggest the need for further enhancements of public health and preventive policies tailored to each gender in Saudi Arabia.

Reducing the burden attributable to risk factors requires identifying the broader organization of associated factors, which will facilitate the establishment of more targeted interventions for both genders. The data show that high body mass indices have increased significantly for both males and females, with notable differences, remaining as the leading risk factor, and the percentage change in DALYs from 1990 to 2021 for both males and females increased. The combination of population aging and significant lifestyle changes led to the high prevalence of overweight and obesity in the Saudi population, which reached 58%, with males having a higher overweight prevalence (43% vs. 33%) and females having a higher obesity prevalence (19% vs. 21) [37,38,39,40]; this was also documented at the global level [41]. Other metabolic factors, including high blood pressure, LDL, and fasting plasma glucose, all increased significantly, as reflected in the increased rate of change in SEVs between 1990 and 2021. Metabolic risk factors are linked to high BMIs and are known to be among the leading risk factors for morbidity and mortality globally and in Saudi Arabia [36,42,43,44]. However, many policies and interventions were implemented that directly or indirectly address risk factors, primarily focusing on obesity and diet [36,45]. Nonetheless, significant challenges remain, such as the lack of information or data related to pre- and post-evaluation interventions that need to be implemented to assess their impact [36].

Furthermore, certain metabolic risk factors, including BMI and blood pressure, are linked to behavioral patterns, such as dietary habits [46]. Rapid urbanization has shifted towards diets low in fruits and vegetables and high in fat, sugar, and highly refined foods, as evidenced by the rate of change in SEVs for dietary habits between 1990 and 2021. The 2013 Saudi Health Interview Survey (SHIS) found that the population consumes a low amount of fruit and vegetables for all age groups, with minor gender differences existing in age groups of 15–24 and 65+ years, where women consumed more. These findings were also reported in a 2017 household survey, where only 10.4% indicated that they consumed vegetables and fruits, with no significant gender variation [36,47]. Another behavioral risk factor is low physical activity, which was among the leading risk factors in females only and was attributed to the burden of several diseases. Globally, the prevalence of physical inactivity is 20% lower for males than females (45% vs. 65%) [18,48]. This shows a similar trend in Saudi Arabia, where the rates of physical activity of males vs. females for at least 150 min per week reached 36.1% and 20.3%, respectively [49,50,51,52,53,54]. However, the recent results of the National Health Survey showed that 58.5% of the Saudi Arabian population (18 years and over) engage in physical activity for 150 min or more per week, with percentages for males vs. females being 64% vs. 46.3%, respectively [55]. This improvement reflects the progress in targeting this risk factor. More than 44 policies/initiatives that span different sectors, such as health, education, sports, tourism, and urban design, have been developed to enhance physical activities [56], such as launching the Quality of Life (QOL) Program in 2020 aimed at improving the population’s ‘livability’ and ‘lifestyles’ to meet one of the primary specified program objectives, namely ‘to increase public participation in sports and athletic activities.’ [57]. Still, insufficient physical activity is a significant risk factor with notable variations between the two genders.

Smoking is another behavioral factor, and despite the aggressive national initiatives to control it, such as a significant increase in tobacco taxes to 100% and the expansion of the smoking ban in public places [58], it was the fifth leading risk factor in males in 2021. Males have a more significant increase in age-standardized rates than women, as documented in the literature [31,59,60,61,62,63]. In 2022, the gender difference in using tobacco reached a stark variation, with rates of 26.3% for males and only 1.9% for females [64]. This variation was linked to several differences in culture and norms of practices [61]. However, more studies are warranted to evaluate the current intervention and investigate the available challenges and variations.

Intimate partner violence (IPV) is a risk factor that was only observed among females and was associated with different disease burdens. Calculated from several regions with different socioeconomic characteristics [65,66], it was estimated that the lifetime prevalence of IPV varies from 35% to 45%, with higher values associated with lower social and economic statuses [65,67,68]. In 2013, the Law for Protection from Abuse was approved [69] to ensure protection from all forms of abuse, provide assistance, services, and shelter, and implement legal proceedings against violators [69]. However, intimate partner violence is a global phenomenon, and several studies have indicated that women’s empowerment in transforming the cultural perceptions of violence is vital in reducing its effects [68,70,71].

Although some behavioral risk factors showed no statistically significant difference in SEV or DALYs, the values indicate a notable public health concern. In other words, these findings underscore the substantial health challenges that women face regarding behavioral risk factors.

Most environmental and occupational risk factors associated with disease burden have decreased for both genders over the years, which is an observation that has also been reported on a global level. Furthermore, an inconsistent relationship was observed between decreasing DALYs and increasing SEVs in this group of risk factors, including air pollution and non-optimal temperature. This might be related to the adoption of sustainable development for the environment, as well as improved healthcare, which both offset risk exposure [2,17,72]. However, Saudi Arabia heavily relies on oil and gas, and based on the results, air pollution, safe drinking water, sanitation, and hygiene (WASH), as well as other environmental factors, remained the top health risks for Saudi Arabia in 2021. This was observed in the increased rate of change for air pollution and non-optimal temperature SEVs for both genders, which is an observation with the same directional relationship at the global level [2]. However, efforts to protect the environment and its resources, such as the launch of the Energy and Environment Research Fund (EERF) project and the Saudi Green Initiative (SGI), have been implemented, resulting in a slight decline in associated DALYs. Nevertheless, to address environmental risk factors, strategies must be implemented at multiple levels, including individual and community levels, to achieve a healthier environment.

Finally, the results of this study should be viewed in consideration of their context and limitations. Initially, this study shares the overarching limitations of the GBD study, which are thoroughly detailed in other sources, such as all the biases in the reported data [73]. Furthermore, in some cases, the 95% UI was large, which may be attributed to limited data availability, small sample sizes, or conflicting data. Moreover, the GBD study provides a comprehensive estimate, although the exact link between an identified risk factor and the related disease outcome may be too complex to precisely quantify. Furthermore, for comparisons, the data were disaggregated by gender; however, the causes of health disparities may be associated with other factors, such as societal norms. Therefore, more in-depth research that considers other confounders is needed to compare the impact of gender, health, and other social, commercial, and digital determinants for a more nuanced understanding of priorities. As evidence continues to accumulate, additional population-specific characteristics will likely be identified.

## 5. Conclusions

With the rapid modernization and the adoption of new lifestyle habits, there has been a significant shift over the past 30 years, resulting in a steady increase in specific risk factors related to disease burden. Examining how disease burden is linked to these risk factors can help guide strategy development. The findings revealed ongoing health disparities between males and females, highlighting the urgent need for gender-specific research, policies, and interventions. Strategies focused on improving health and reducing disease burden should consider the unique health challenges faced by different genders.

## Data Availability

All citations and metadata used in this study are available in the (GBD 2021) Sources Tool.

## Supplementary Materials

The following supporting information can be downloaded at: https://www.mdpi.com/article/10.3390/healthcare13141717/s1, Table S1: Age standardised SEVs in1990, and 2021, and rate of change over 1990- 2021 by GBD level two risk factor; Figure S1: Leading risk factors by attributable DALYs (per 100.000) for all age, and percent change in DALYs from 1990 to 2021 in Saudi Arabia; Figure S2: Age-standardized DALYs per 100,000 for Level 2 risk factors by cause in males and females in Saudi Arabia, 2021; Figure S3: Percentage of Death attributable to Level two risk factors by cause and sex in Saudi Arabia 2021.

## Abbreviations

GBD	Global burden of disease
DALYs	Disability-adjusted life years
HALE	Healthy life expectancy at birth
SEV	Summary exposure value
GATHER	Guidelines for Accurate and Transparent Health Estimates Reporting
YLL	Years of life lost
YLD	Years lived with disability
