# Supplementary material for: Three Decades of Trends in Risk Factors Attributed to Disease Burden in Saudi Arabia: Findings from the Global Burden of Disease Study 2021
Source: Healthcare (Basel). 2025 Jul 17;13(14):1717. doi: 10.3390/healthcare13141717 (PMC12295640; doi:10.3390/healthcare13141717)
Supplement: Supplementary file 1 [file healthcare-13-01717-s001.zip › healthcare-3694207-supplementary.pdf]

Table S1: Age standardised SEVs in1990, and 2021, and rate of change over 1990- 2021 by GBD level two risk factor

sex_name	RF_name	year	val	upper	lower
Male	Unsafe water, sanitation, and handwashing	1990	23.0321	32.00193	12.86645
Female	Unsafe water, sanitation, and handwashing	1990	23.0321	32.00193	12.86645
Male	Air pollution	1990	39.2868	48.33069	30.44731
Female	Air pollution	1990	37.79141	46.77585	29.20965
Male	Other environmental risks	1990	52.12856	62.89878	2.884659
Female	Other environmental risks	1990	35.87041	45.31808	2.884659
Male	Child and maternal malnutrition	1990	7.36786	12.88115	3.895335
Female	Child and maternal malnutrition	1990	12.73473	18.18656	8.270961
Male	Tobacco	1990	32.42498	36.28956	28.75813
Female	Tobacco	1990	18.9393	21.14663	16.74969
Male	High alcohol use	1990	1.36931	4.304873	0.512619
Female	High alcohol use	1990	0.494317	1.485359	0.156758
Male	Drug use	1990	0.346982	0.483549	0.216557
Female	Drug use	1990	0.494931	0.755457	0.226299
Male	High fasting plasma glucose	1990	17.24966	21.0767	13.29808
Female	High fasting plasma glucose	1990	14.0979	17.2629	10.31561
Male	High systolic blood pressure	1990	36.03259	51.22767	24.08902
Female	High systolic blood pressure	1990	33.82052	49.93252	20.84292
Male	High body-mass index	1990	23.27126	27.0701	20.15954
Female	High body-mass index	1990	29.70602	33.75597	25.31554
Male	Low bone mineral density	1990	20.80656	28.28124	14.52269
Female	Low bone mineral density	1990	27.14542	35.08555	20.13576
Male	Dietary risks	1990	28.65609	39.37224	19.58102
Female	Dietary risks	1990	27.64626	36.39331	19.08401
Male	Low physical activity	1990	20.82583	26.5909	15.47082
Female	Low physical activity	1990	35.28403	42.50798	28.79588
Male	Occupational risks	1990	3.978262	4.557402	3.452078

Female	Occupational risks	1990	0.489208	0.645957	0.372144
Female	Intimate partner violence	1990	24.05315	31.71381	13.65586
Male	Non-optimal temperature	1990	79.62376	87.38468	70.78182
Female	Non-optimal temperature	1990	79.62376	87.38468	70.78182
Male	Kidney dysfunction	1990	2.954388	3.894586	2.323796
Female	Kidney dysfunction	1990	3.001107	3.945322	2.39426
Male	High LDL cholesterol	1990	38.83158	55.24607	25.39466
Female	High LDL cholesterol	1990	36.18968	51.78189	23.58126
Male	Childhood sexual abuse and bullying	1990	7.150837	12.47206	3.846256
Female	Childhood sexual abuse and bullying	1990	4.969216	7.311835	3.555448
Male	Unsafe water, sanitation, and handwashing	2021	5.545614	8.508341	2.590389
Female	Unsafe water, sanitation, and handwashing	2021	5.545614	8.508341	2.590389
Male	Air pollution	2021	42.93965	51.19271	35.1801
Female	Air pollution	2021	41.19621	49.32935	33.6768
Male	Other environmental risks	2021	32.08232	39.5064	2.52132
Female	Other environmental risks	2021	23.31467	29.66681	2.50541
Male	Child and maternal malnutrition	2021	3.29836	5.79033	1.802342
Female	Child and maternal malnutrition	2021	11.95892	16.70424	7.927736
Male	Tobacco	2021	32.79717	36.35997	29.20773
Female	Tobacco	2021	21.06555	23.55907	18.69605
Male	High alcohol use	2021	0.993569	3.285593	0.343601
Female	High alcohol use	2021	0.405564	1.283462	0.126301
Male	Drug use	2021	0.328793	0.44456	0.212875
Female	Drug use	2021	0.513459	0.761089	0.223908
Male	High fasting plasma glucose	2021	29.74473	35.81789	22.52503
Female	High fasting plasma glucose	2021	30.27047	37.12191	21.86173
Male	High systolic blood pressure	2021	35.72537	49.0014	24.58636
Female	High systolic blood pressure	2021	22.17789	33.48882	14.02543
Male	High body-mass index	2021	50.75978	57.10808	42.28968

Female	High body-mass index	2021	57.98278	64.07476	49.2217
Male	Low bone mineral density	2021	17.96873	25.14827	11.63977
Female	Low bone mineral density	2021	23.57814	30.79791	17.0942
Male	Dietary risks	2021	31.7635	42.60167	22.10316
Female	Dietary risks	2021	31.29455	40.6444	22.8564
Male	Low physical activity	2021	26.98384	34.29819	20.49743
Female	Low physical activity	2021	46.32115	54.89234	38.23768
Male	Occupational risks	2021	3.843292	4.460734	3.350356
Female	Occupational risks	2021	0.764746	0.967481	0.622972
Female	Intimate partner violence	2021	23.99252	31.3702	13.60517
Male	Non-optimal temperature	2021	86.50595	94.99007	74.73818
Female	Non-optimal temperature	2021	86.50595	94.99007	74.73818
Male	Kidney dysfunction	2021	2.949655	3.959617	2.308774
Female	Kidney dysfunction	2021	3.027239	3.932585	2.451665
Male	High LDL cholesterol	2021	47.78897	66.72929	32.09912
Female	High LDL cholesterol	2021	46.94815	65.68836	31.99276
Male	Childhood sexual abuse and bullying	2021	8.847671	16.19818	4.260666
Female	Childhood sexual abuse and bullying	2021	6.499085	10.45396	4.10039

Figure S1: Leading risk factors by attributable DALYs (per 100.000) for all age, and percent change in DALYs from 1990 to 2021 in Saudi Arabia

Year	Sex	Cause of death or injury	Risk factor	Measure	Value	Lower bound	Upper bound
1990	Male	All causes	Unsafe water, sanitation, and handwashing	DALY rank	12		
1990	Male	All causes	Unsafe water, sanitation, and handwashing	DALYs per 100,000	786.1226	416.512765	1256.76421
2021	Male	All causes	Unsafe water, sanitation, and handwashing	DALY rank	19		
2021	Male	All causes	Unsafe water, sanitation, and handwashing	DALYs per 100,000	45.2952837	17.5219637	80.3561648
1990 - 2021	Male	All causes	Unsafe water, sanitation, and handwashing	DALYs % change	-0.9423814		
1990	Male	All causes	Air pollution	DALY rank	2		

1990	Male	All causes	Air pollution	DALYs per 100,000	2808.03289	1929.48936	3932.32422
2021	Male	All causes	Air pollution	DALY rank	6		
2021	Male	All causes	Air pollution	DALYs per 100,000	2354.97167	1744.46151	3022.37978
1990 - 2021	Male	All causes	Air pollution	DALYs % change	-0.1613447		
1990	Male	All causes	Other environmental risks	DALY rank	13		
1990	Male	All causes	Other environmental risks	DALYs per 100,000	506.932169	-33.440172	1070.66807
2021	Male	All causes	Other environmental risks	DALY rank	11		
2021	Male	All causes	Other environmental risks	DALYs per 100,000	461.29374	-28.251631	943.328103
1990 - 2021	Male	All causes	Other environmental risks	DALYs % change	-0.0900287		
1990	Male	All causes	Child and maternal malnutrition	DALY rank	1		
1990	Male	All causes	Child and maternal malnutrition	DALYs per 100,000	6791.80304	5355.14447	8510.80299
2021	Male	All causes	Child and maternal malnutrition	DALY rank	12		
2021	Male	All causes	Child and maternal malnutrition	DALYs per 100,000	375.942679	297.535254	463.660144
1990 - 2021	Male	All causes	Child and maternal malnutrition	DALYs % change	-0.9446476		
1990	Male	All causes	Tobacco	DALY rank	5		
1990	Male	All causes	Tobacco	DALYs per 100,000	1568.51329	1071.72007	2148.07683
2021	Male	All causes	Tobacco	DALY rank	5		
2021	Male	All causes	Tobacco	DALYs per 100,000	2359.74075	1685.86636	3102.75296
1990 - 2021	Male	All causes	Tobacco	DALYs % change	0.50444422		
1990	Male	All causes	High alcohol use	DALY rank	15		
1990	Male	All causes	High alcohol use	DALYs per 100,000	161.962578	97.6528019	245.329244
2021	Male	All causes	High alcohol use	DALY rank	17		
2021	Male	All causes	High alcohol use	DALYs per 100,000	143.816318	96.3780971	202.694611
1990 - 2021	Male	All causes	High alcohol use	DALYs % change	-0.1120398		
1990	Male	All causes	Drug use	DALY rank	16		
1990	Male	All causes	Drug use	DALYs per 100,000	133.52976	93.946534	187.455036
2021	Male	All causes	Drug use	DALY rank	14		

2021	Male	All causes	Drug use	DALYs per 100,000	254.337806	177.239941	356.860573
1990 - 2021	Male	All causes	Drug use	DALYs % change	0.9047275		
1990	Male	All causes	High fasting plasma glucose	DALY rank	9		
1990	Male	All causes	High fasting plasma glucose	DALYs per 100,000	1048.67607	827.282807	1292.38859
2021	Male	All causes	High fasting plasma glucose	DALY rank	4		
2021	Male	All causes	High fasting plasma glucose	DALYs per 100,000	2374.0495	1929.4416	2871.75329
1990 - 2021	Male	All causes	High fasting plasma glucose	DALYs % change	1.26385398		
1990	Male	All causes	High systolic blood pressure	DALY rank	3		
1990	Male	All causes	High systolic blood pressure	DALYs per 100,000	2201.32097	1587.26798	2925.36825
2021	Male	All causes	High systolic blood pressure	DALY rank	2		
2021	Male	All causes	High systolic blood pressure	DALYs per 100,000	3022.87149	2270.04396	3826.8981
1990 - 2021	Male	All causes	High systolic blood pressure	DALYs % change	0.37320796		
1990	Male	All causes	High body-mass index	DALY rank	6		
1990	Male	All causes	High body-mass index	DALYs per 100,000	1280.3143	641.480114	2037.42141
2021	Male	All causes	High body-mass index	DALY rank	1		
2021	Male	All causes	High body-mass index	DALYs per 100,000	3436.22799	1878.71088	5031.46521
1990 - 2021	Male	All causes	High body-mass index	DALYs % change	1.6838941		
1990	Male	All causes	Low bone mineral density	DALY rank	14		
1990	Male	All causes	Low bone mineral density	DALYs per 100,000	256.691922	200.969561	317.869577
2021	Male	All causes	Low bone mineral density	DALY rank	13		
2021	Male	All causes	Low bone mineral density	DALYs per 100,000	359.487651	281.831976	444.835087
1990 - 2021	Male	All causes	Low bone mineral density	DALYs % change	0.40046343		
1990	Male	All causes	Dietary risks	DALY rank	4		
1990	Male	All causes	Dietary risks	DALYs per 100,000	1698.23431	153.290508	2624.86611
2021	Male	All causes	Dietary risks	DALY rank	3		
2021	Male	All causes	Dietary risks	DALYs per 100,000	2922.38454	268.084049	4491.08146
1990 - 2021	Male	All causes	Dietary risks	DALYs % change	0.72083707		

1990	Male	All causes	Low physical activity	DALY rank	18		
1990	Male	All causes	Low physical activity	DALYs per 100,000	123.770859	55.3539379	213.560878
2021	Male	All causes	Low physical activity	DALY rank	15		
2021	Male	All causes	Low physical activity	DALYs per 100,000	231.133271	97.6445061	374.337307
1990 - 2021	Male	All causes	Low physical activity	DALYs % change	0.86742884		
1990	Male	All causes	Occupational risks	DALY rank	10		
1990	Male	All causes	Occupational risks	DALYs per 100,000	1036.35396	850.238884	1232.8394
2021	Male	All causes	Occupational risks	DALY rank	10		
2021	Male	All causes	Occupational risks	DALYs per 100,000	676.979202	545.382408	826.435937
1990 - 2021	Male	All causes	Occupational risks	DALYs % change	-0.3467684		
1990	Male	All causes	Intimate partner violence	DALY rank			
1990	Male	All causes	Intimate partner violence	DALYs per 100,000			
2021	Male	All causes	Intimate partner violence	DALY rank			
2021	Male	All causes	Intimate partner violence	DALYs per 100,000			
1990 - 2021	Male	All causes	Intimate partner violence	DALYs % change	0		
1990	Male	All causes	Unsafe sex	DALY rank	19		
1990	Male	All causes	Unsafe sex	DALYs per 100,000	18.8889348	15.0247921	23.358748
2021	Male	All causes	Unsafe sex	DALY rank	18		
2021	Male	All causes	Unsafe sex	DALYs per 100,000	52.4195909	45.6396464	59.9884437
1990 - 2021	Male	All causes	Unsafe sex	DALYs % change	1.77514807		
1990	Male	All causes	Non-optimal temperature	DALY rank	7		
1990	Male	All causes	Non-optimal temperature	DALYs per 100,000	1081.74118	624.909153	1573.89034
2021	Male	All causes	Non-optimal temperature	DALY rank	9		
2021	Male	All causes	Non-optimal temperature	DALYs per 100,000	1335.82425	752.281027	1961.3776
1990 - 2021	Male	All causes	Non-optimal temperature	DALYs % change	0.23488342		
1990	Male	All causes	Kidney dysfunction	DALY rank	11		
1990	Male	All causes	Kidney dysfunction	DALYs per 100,000	930.143361	699.175107	1184.56971

2021	Male	All causes	Kidney dysfunction	DALY rank	8		
2021	Male	All causes	Kidney dysfunction	DALYs per 100,000	1608.06595	1207.80725	2013.85537
1990 - 2021	Male	All causes	Kidney dysfunction	DALYs % change	0.72883667		
1990	Male	All causes	High LDL cholesterol	DALY rank	8		
1990	Male	All causes	High LDL cholesterol	DALYs per 100,000	1051.83234	658.033665	1564.04902
2021	Male	All causes	High LDL cholesterol	DALY rank	7		
2021	Male	All causes	High LDL cholesterol	DALYs per 100,000	1982.53906	1356.05581	2750.89946
1990 - 2021	Male	All causes	High LDL cholesterol	DALYs % change	0.88484322		
1990	Male	All causes	Childhood sexual abuse and bullying	DALY rank	17		
1990	Male	All causes	Childhood sexual abuse and bullying	DALYs per 100,000	125.605632	54.5148072	240.322691
2021	Male	All causes	Childhood sexual abuse and bullying	DALY rank	16		
2021	Male	All causes	Childhood sexual abuse and bullying	DALYs per 100,000	177.531615	76.4271412	330.310186
1990 - 2021	Male	All causes	Childhood sexual abuse and bullying	DALYs % change	0.4134049		
1990	Female	All causes	Unsafe water, sanitation, and handwashing	DALY rank	9		
1990	Female	All causes	Unsafe water, sanitation, and handwashing	DALYs per 100,000	740.503845	374.661297	1206.46129
2021	Female	All causes	Unsafe water, sanitation, and handwashing	DALY rank	19		
2021	Female	All causes	Unsafe water, sanitation, and handwashing	DALYs per 100,000	55.7782102	21.774309	98.8604098
1990 - 2021	Female	All causes	Unsafe water, sanitation, and handwashing	DALYs % change	-0.9246753		
1990	Female	All causes	Air pollution	DALY rank	2		
1990	Female	All causes	Air pollution	DALYs per 100,000	2366.22267	1619.88984	3238.9397
2021	Female	All causes	Air pollution	DALY rank	5		
2021	Female	All causes	Air pollution	DALYs per 100,000	1614.75559	1203.81813	2065.24776
1990 - 2021	Female	All causes	Air pollution	DALYs % change	-0.3175809		
1990	Female	All causes	Other environmental risks	DALY rank	12		
1990	Female	All causes	Other environmental risks	DALYs per 100,000	292.814587	-15.019473	637.212773
2021	Female	All causes	Other environmental risks	DALY rank	13		
2021	Female	All causes	Other environmental risks	DALYs per 100,000	225.058854	-11.646411	476.599591

1990 - 2021	Female	All causes	Other environmental risks	DALYs % change	-0.2313947		
1990	Female	All causes	Child and maternal malnutrition	DALY rank	1		
1990	Female	All causes	Child and maternal malnutrition	DALYs per 100,000	6347.17288	5135.31918	7841.98664
2021	Female	All causes	Child and maternal malnutrition	DALY rank	9		
2021	Female	All causes	Child and maternal malnutrition	DALYs per 100,000	740.439228	556.513621	959.694157
1990 - 2021	Female	All causes	Child and maternal malnutrition	DALYs % change	-0.8833435		
1990	Female	All causes	Tobacco	DALY rank	11		
1990	Female	All causes	Tobacco	DALYs per 100,000	495.568248	278.734326	766.596302
2021	Female	All causes	Tobacco	DALY rank	10		
2021	Female	All causes	Tobacco	DALYs per 100,000	541.766818	315.552977	779.27005
1990 - 2021	Female	All causes	Tobacco	DALYs % change	0.09322343		
1990	Female	All causes	High alcohol use	DALY rank	20		
1990	Female	All causes	High alcohol use	DALYs per 100,000	33.7052045	22.1340898	50.3371949
2021	Female	All causes	High alcohol use	DALY rank	20		
2021	Female	All causes	High alcohol use	DALYs per 100,000	35.1630532	24.6545905	48.0208511
1990 - 2021	Female	All causes	High alcohol use	DALYs % change	0.04325293		
1990	Female	All causes	Drug use	DALY rank	16		
1990	Female	All causes	Drug use	DALYs per 100,000	106.285804	70.3671743	143.169133
2021	Female	All causes	Drug use	DALY rank	14		
2021	Female	All causes	Drug use	DALYs per 100,000	162.140479	116.906298	215.197654
1990 - 2021	Female	All causes	Drug use	DALYs % change	0.52551398		
1990	Female	All causes	High fasting plasma glucose	DALY rank	7		
1990	Female	All causes	High fasting plasma glucose	DALYs per 100,000	876.676153	682.601328	1104.41227
2021	Female	All causes	High fasting plasma glucose	DALY rank	2		
2021	Female	All causes	High fasting plasma glucose	DALYs per 100,000	2003.67359	1615.2059	2468.4295
1990 - 2021	Female	All causes	High fasting plasma glucose	DALYs % change	1.2855345		
1990	Female	All causes	High systolic blood pressure	DALY rank	3		

1990	Female	All causes	High systolic blood pressure	DALYs per 100,000	1838.3066	1329.93832	2463.99929
2021	Female	All causes	High systolic blood pressure	DALY rank	4		
2021	Female	All causes	High systolic blood pressure	DALYs per 100,000	1734.09261	1245.57989	2264.37559
1990 - 2021	Female	All causes	High systolic blood pressure	DALYs % change	-0.0566902		
1990	Female	All causes	High body-mass index	DALY rank	4		
1990	Female	All causes	High body-mass index	DALYs per 100,000	1311.42734	606.565562	2067.00661
2021	Female	All causes	High body-mass index	DALY rank	1		
2021	Female	All causes	High body-mass index	DALYs per 100,000	2952.61908	1456.96768	4407.00432
1990 - 2021	Female	All causes	High body-mass index	DALYs % change	1.25145457		
1990	Female	All causes	Low bone mineral density	DALY rank	15		
1990	Female	All causes	Low bone mineral density	DALYs per 100,000	125.481737	98.6080372	154.879984
2021	Female	All causes	Low bone mineral density	DALY rank	15		
2021	Female	All causes	Low bone mineral density	DALYs per 100,000	157.34632	121.859647	198.1603
1990 - 2021	Female	All causes	Low bone mineral density	DALYs % change	0.25393802		
1990	Female	All causes	Dietary risks	DALY rank	5		
1990	Female	All causes	Dietary risks	DALYs per 100,000	1250.50302	59.0053522	1960.10938
2021	Female	All causes	Dietary risks	DALY rank	3		
2021	Female	All causes	Dietary risks	DALYs per 100,000	1877.87438	198.28277	2909.9177
1990 - 2021	Female	All causes	Dietary risks	DALYs % change	0.5016952		
1990	Female	All causes	Low physical activity	DALY rank	14		
1990	Female	All causes	Low physical activity	DALYs per 100,000	189.377391	85.9460469	310.430108
2021	Female	All causes	Low physical activity	DALY rank	11		
2021	Female	All causes	Low physical activity	DALYs per 100,000	327.985037	138.472823	528.518076
1990 - 2021	Female	All causes	Low physical activity	DALYs % change	0.73191232		
1990	Female	All causes	Occupational risks	DALY rank	18		
1990	Female	All causes	Occupational risks	DALYs per 100,000	56.7182996	41.9648289	74.2138695
2021	Female	All causes	Occupational risks	DALY rank	18		

2021	Female	All causes	Occupational risks	DALYs per 100,000	89.4007944	67.5638697	116.143392
1990 - 2021	Female	All causes	Occupational risks	DALYs % change	0.57622487		
1990	Female	All causes	Intimate partner violence	DALY rank	13		
1990	Female	All causes	Intimate partner violence	DALYs per 100,000	192.900105	110.590914	293.633735
2021	Female	All causes	Intimate partner violence	DALY rank	12		
2021	Female	All causes	Intimate partner violence	DALYs per 100,000	254.525939	120.967315	423.61301
1990 - 2021	Female	All causes	Intimate partner violence	DALYs % change	0.31947019		
1990	Female	All causes	Unsafe sex	DALY rank	19		
1990	Female	All causes	Unsafe sex	DALYs per 100,000	55.831524	47.0885429	68.7503896
2021	Female	All causes	Unsafe sex	DALY rank	16		
2021	Female	All causes	Unsafe sex	DALYs per 100,000	141.869902	128.660591	165.029792
1990 - 2021	Female	All causes	Unsafe sex	DALYs % change	1.54103582		
1990	Female	All causes	Non-optimal temperature	DALY rank	6		
1990	Female	All causes	Non-optimal temperature	DALYs per 100,000	944.995134	590.857294	1348.67109
2021	Female	All causes	Non-optimal temperature	DALY rank	8		
2021	Female	All causes	Non-optimal temperature	DALYs per 100,000	861.333612	563.55986	1220.57184
1990 - 2021	Female	All causes	Non-optimal temperature	DALYs % change	-0.0885312		
1990	Female	All causes	Kidney dysfunction	DALY rank	8		
1990	Female	All causes	Kidney dysfunction	DALYs per 100,000	832.029048	641.975694	1056.4484
2021	Female	All causes	Kidney dysfunction	DALY rank	6		
2021	Female	All causes	Kidney dysfunction	DALYs per 100,000	1558.62685	1205.57568	1966.37439
1990 - 2021	Female	All causes	Kidney dysfunction	DALYs % change	0.87328418		
1990	Female	All causes	High LDL cholesterol	DALY rank	10		
1990	Female	All causes	High LDL cholesterol	DALYs per 100,000	719.88304	423.11148	1060.80001
2021	Female	All causes	High LDL cholesterol	DALY rank	7		
2021	Female	All causes	High LDL cholesterol	DALYs per 100,000	1149.24648	761.455743	1628.84613
1990 - 2021	Female	All causes	High LDL cholesterol	DALYs % change	0.59643499		

1990	Female	All causes	Childhood sexual abuse and bullying	DALY rank	17		
1990	Female	All causes	Childhood sexual abuse and bullying	DALYs per 100,000	77.8793894	37.6254245	154.948641
2021	Female	All causes	Childhood sexual abuse and bullying	DALY rank	17		
2021	Female	All causes	Childhood sexual abuse and bullying	DALYs per 100,000	135.750307	63.1441535	257.977198
1990 - 2021	Female	All causes	Childhood sexual abuse and bullying	DALYs % change	0.74308386		

Figure S2: Age-standardized DALYs per 100,000 for Level 2 risk factors by cause in males and females in Saudi Arabia, 2021.

measure_name	sex_name	age_name	cause_name	rei_name	val	upper	lower
DALYs	Male	Age-standardized	Non-communicable diseases	Suboptimal temperature	1462.78953	2016.67524	930.476438
DALYs	Female	Age-standardized	Non-communicable diseases	Suboptimal temperature	1254.37988	1763.09317	784.513108
DALYs	Male	Age-standardized	Non-communicable diseases	Impaired kidney function	2554.64247	3081.66983	1984.43498
DALYs	Female	Age-standardized	Non-communicable diseases	Impaired kidney function	2612.14007	3241.33301	2063.05355
DALYs	Male	Age-standardized	Non-communicable diseases	Childhood maltreatment	152.833876	275.715355	68.060956
DALYs	Female	Age-standardized	Non-communicable diseases	Childhood maltreatment	120.848012	231.125613	56.7547827
DALYs	Male	Age-standardized	Non-communicable diseases	High LDL cholesterol	2297.87378	3159.77422	1490.56158
DALYs	Female	Age-standardized	Non-communicable diseases	High LDL cholesterol	1667.10145	2402.45271	1023.13246
DALYs	Male	Age-standardized	Injuries	Alcohol use	19.6218862	35.7083343	5.30382714
DALYs	Female	Age-standardized	Injuries	Alcohol use	0.16596022	0.35693537	0.01758704
DALYs	Male	Age-standardized	Injuries	Occupational risks	232.099431	292.592474	174.950184
DALYs	Female	Age-standardized	Injuries	Occupational risks	8.47019126	11.396313	6.41237142
DALYs	Male	Age-standardized	Injuries	Low bone mineral density	454.051561	546.094442	368.598423
DALYs	Female	Age-standardized	Injuries	Low bone mineral density	262.43658	327.427532	205.907955
DALYs	Male	Age-standardized	Injuries	Tobacco	26.9100591	36.057638	19.2047015
DALYs	Female	Age-standardized	Injuries	Tobacco	1.85885324	2.7361018	1.14302936

DALYs	Male	Age-standardized	Injuries	Drug use	5.97297536	14.9345451	1.45733879
DALYs	Female	Age-standardized	Injuries	Drug use	3.23287871	8.34300637	0.85941171
DALYs	Male	Age-standardized	Communicable, maternal, neonatal, and nutritional diseases	Unsafe water, sanitation, and handwashing	66.0188807	117.706039	25.5926018
DALYs	Female	Age-standardized	Communicable, maternal, neonatal, and nutritional diseases	Unsafe water, sanitation, and handwashing	76.75063	136.395957	29.9670087
DALYs	Male	Age-standardized	Communicable, maternal, neonatal, and nutritional diseases	Air pollution	253.302738	413.700781	91.2986048
DALYs	Female	Age-standardized	Communicable, maternal, neonatal, and nutritional diseases	Air pollution	228.263814	382.503639	69.9626481
DALYs	Male	Age-standardized	Communicable, maternal, neonatal, and nutritional diseases	Child and maternal malnutrition	549.541981	666.721745	434.459814
DALYs	Female	Age-standardized	Communicable, maternal, neonatal, and nutritional diseases	Child and maternal malnutrition	796.21034	1007.24995	610.217445
DALYs	Male	Age-standardized	Communicable, maternal, neonatal, and nutritional diseases	Drug use	43.6139989	47.3511904	40.450379
DALYs	Female	Age-standardized	Communicable, maternal, neonatal, and nutritional diseases	Drug use	11.8149407	14.5628137	9.58347872
DALYs	Male	Age-standardized	Non-communicable diseases	Air pollution	3218.80775	3943.87338	2503.72103
DALYs	Female	Age-standardized	Non-communicable diseases	Air pollution	2592.98724	3245.25188	1928.26129
DALYs	Male	Age-standardized	Non-communicable diseases	Drug use	150.892129	234.391489	83.3889546
DALYs	Female	Age-standardized	Non-communicable diseases	Drug use	115.22424	156.49608	78.4587001
DALYs	Male	Age-standardized	Communicable, maternal, neonatal, and nutritional diseases	Tobacco	156.33462	224.015858	96.9129182
DALYs	Female	Age-standardized	Communicable, maternal, neonatal, and nutritional diseases	Tobacco	61.6504929	105.284607	24.0572969
DALYs	Female	Age-standardized	Injuries	Intimate partner violence	105.582345	151.210548	68.8480615
DALYs	Male	Age-standardized	Non-communicable diseases	High body-mass index	4579.41592	6594.74896	2513.2396
DALYs	Female	Age-standardized	Non-communicable diseases	High body-mass index	4509.36342	6747.95951	2309.49191
DALYs	Male	Age-standardized	Non-communicable diseases	Occupational risks	354.938748	439.925881	277.79333
DALYs	Female	Age-standardized	Non-communicable diseases	Occupational risks	65.528888	84.4550518	48.6860896
DALYs	Male	Age-standardized	Communicable, maternal, neonatal, and nutritional diseases	Alcohol use	2.29774081	15.1852458	-0.8699611

DALYs	Female	Age-standardized	Communicable, maternal, neonatal, and nutritional diseases	Alcohol use	0.07905316	0.46276876	-0.0393938
DALYs	Male	Age-standardized	Communicable, maternal, neonatal, and nutritional diseases	High body-mass index	28.2170107	67.945675	6.23707372
DALYs	Female	Age-standardized	Communicable, maternal, neonatal, and nutritional diseases	High body-mass index	38.4386293	80.2488975	10.5777852
DALYs	Male	Age-standardized	Communicable, maternal, neonatal, and nutritional diseases	Dietary risks	4.31056324	9.25075056	1.2011911
DALYs	Female	Age-standardized	Communicable, maternal, neonatal, and nutritional diseases	Dietary risks	5.86374014	11.317561	1.70532574
DALYs	Male	Age-standardized	Non-communicable diseases	High systolic blood pressure	5113.21001	6099.63635	4103.76869
DALYs	Female	Age-standardized	Non-communicable diseases	High systolic blood pressure	3704.48627	4675.94161	2801.00789
DALYs	Female	Age-standardized	Communicable, maternal, neonatal, and nutritional diseases	Intimate partner violence	8.17600975	12.992601	4.06648264
DALYs	Male	Age-standardized	Communicable, maternal, neonatal, and nutritional diseases	Unsafe sex	38.512229	43.7886435	33.6212336
DALYs	Female	Age-standardized	Communicable, maternal, neonatal, and nutritional diseases	Unsafe sex	86.287422	98.9953013	78.3624875
DALYs	Female	Age-standardized	Non-communicable diseases	Unsafe sex	29.9864868	40.5939207	22.0590659
DALYs	Male	Age-standardized	Injuries	Suboptimal temperature	225.626847	519.318962	-114.95818
DALYs	Female	Age-standardized	Injuries	Suboptimal temperature	63.9197953	155.881631	-37.323739
DALYs	Male	Age-standardized	Non-communicable diseases	Low physical activity	397.246377	644.3838	172.880703
DALYs	Female	Age-standardized	Non-communicable diseases	Low physical activity	567.50456	895.616486	229.160657
DALYs	Male	Age-standardized	Communicable, maternal, neonatal, and nutritional diseases	Suboptimal temperature	124.096329	200.172797	23.9416316
DALYs	Female	Age-standardized	Communicable, maternal, neonatal, and nutritional diseases	Suboptimal temperature	116.281475	202.03955	26.655826
DALYs	Female	Age-standardized	Non-communicable diseases	Intimate partner violence	92.7047576	217.332024	0.37619811
DALYs	Male	Age-standardized	Communicable, maternal, neonatal, and nutritional diseases	Low physical activity	2.26066609	5.02598232	0.62457265
DALYs	Female	Age-standardized	Communicable, maternal, neonatal, and nutritional diseases	Low physical activity	3.35519856	6.73550113	0.98107054
DALYs	Male	Age-standardized	Non-communicable diseases	Other environmental risks	877.532892	1802.38247	-66.196416
DALYs	Female	Age-standardized	Non-communicable diseases	Other environmental risks	517.284369	1091.07114	-39.520002

DALYs	Male	Age-standardized	Non-communicable diseases	Child and maternal malnutrition	1.09849827	2.41978487	0.3069898
DALYs	Female	Age-standardized	Non-communicable diseases	Child and maternal malnutrition	0.79549086	1.73224391	0.22272765
DALYs	Male	Age-standardized	Communicable, maternal, neonatal, and nutritional diseases	High fasting plasma glucose	28.7453081	49.9942948	17.8591898
DALYs	Female	Age-standardized	Communicable, maternal, neonatal, and nutritional diseases	High fasting plasma glucose	27.6629032	40.6812276	18.6537185
DALYs	Male	Age-standardized	Non-communicable diseases	Tobacco	2497.69905	3227.51351	1803.27696
DALYs	Female	Age-standardized	Non-communicable diseases	Tobacco	674.519101	971.061509	413.110339
DALYs	Male	Age-standardized	Non-communicable diseases	Alcohol use	120.125754	160.252183	89.3765331
DALYs	Female	Age-standardized	Non-communicable diseases	Alcohol use	37.4974707	50.1659687	27.6005268
DALYs	Male	Age-standardized	Non-communicable diseases	High fasting plasma glucose	3701.77165	4387.79678	3069.16909
DALYs	Female	Age-standardized	Non-communicable diseases	High fasting plasma glucose	3478.74171	4234.21841	2813.38218
DALYs	Male	Age-standardized	Non-communicable diseases	Dietary risks	3740.73485	5633.13252	573.640452
DALYs	Female	Age-standardized	Non-communicable diseases	Dietary risks	2892.53987	4463.38704	466.876179

Figure S3: Percentage of Death attributable to Level two risk factors by cause and sex in Saudi Arabia 2021

measure	sex	age	cause_name	rei_name	metric	year	val	upper	lower
Deaths	Male	All ages	Non-communicable diseases	High body-mass index	Percent	2021	0.26196356	0.35909982	0.1534633
Deaths	Female	All ages	Non-communicable diseases	High body-mass index	Percent	2021	0.26755164	0.3619399	0.15434965
Deaths	Male	All ages	Communicable, maternal, neonatal, and nutritional diseases	Unsafe water, sanitation, and handwashing	Percent	2021	0.01255023	0.02744757	0.00303066
Deaths	Female	All ages	Communicable, maternal, neonatal, and nutritional diseases	Unsafe water, sanitation, and handwashing	Percent	2021	0.01931858	0.03909598	0.00583741
Deaths	Male	All ages	Non-communicable diseases	Occupational risks	Percent	2021	0.00656158	0.00764579	0.00539484
Deaths	Female	All ages	Non-communicable diseases	Occupational risks	Percent	2021	0.00074067	0.00095726	0.00059477
Deaths	Male	All ages	Communicable, maternal, neonatal, and nutritional diseases	Tobacco	Percent	2021	0.06790243	0.10362552	0.04095983
Deaths	Female	All ages	Communicable, maternal, neonatal, and nutritional diseases	Tobacco	Percent	2021	0.0317631	0.05105467	0.01279847
Deaths	Male	All ages	Non-communicable diseases	Child and maternal malnutrition	Percent	2021	2.18E-05	4.96E-05	6.28E-06

Deaths	Female	All ages	Non-communicable diseases	Child and maternal malnutrition	Percent	2021	2.50E-05	5.36E-05	7.41E-06
Deaths	Male	All ages	Non-communicable diseases	Alcohol use	Percent	2021	0.00461348	0.00667773	0.00303866
Deaths	Female	All ages	Non-communicable diseases	Alcohol use	Percent	2021	0.00135344	0.00187621	0.00093244
Deaths	Male	All ages	Non-communicable diseases	High fasting plasma glucose	Percent	2021	0.18260852	0.21184666	0.15346513
Deaths	Female	All ages	Non-communicable diseases	High fasting plasma glucose	Percent	2021	0.19527851	0.22585691	0.16656179
Deaths	Male	All ages	Non-communicable diseases	Dietary risks	Percent	2021	0.26140393	0.37818283	0.02621537
Deaths	Female	All ages	Non-communicable diseases	Dietary risks	Percent	2021	0.21092153	0.31533648	0.02546474
Deaths	Male	All ages	Communicable, maternal, neonatal, and nutritional diseases	Air pollution	Percent	2021	0.07586141	0.1332956	0.01870978
Deaths	Female	All ages	Communicable, maternal, neonatal, and nutritional diseases	Air pollution	Percent	2021	0.09596978	0.1615433	0.02159538
Deaths	Male	All ages	Non-communicable diseases	High LDL cholesterol	Percent	2021	0.17942114	0.23266739	0.12603381
Deaths	Female	All ages	Non-communicable diseases	High LDL cholesterol	Percent	2021	0.13344082	0.17610002	0.08980016
Deaths	Male	All ages	Communicable, maternal, neonatal, and nutritional diseases	Alcohol use	Percent	2021	0.00104185	0.0068045	-0.0003752
Deaths	Female	All ages	Communicable, maternal, neonatal, and nutritional diseases	Alcohol use	Percent	2021	4.03E-05	0.00023053	-2.03E-05
Deaths	Male	All ages	Communicable, maternal, neonatal, and nutritional diseases	Child and maternal malnutrition	Percent	2021	0.03780359	0.0502688	0.02666197
Deaths	Female	All ages	Communicable, maternal, neonatal, and nutritional diseases	Child and maternal malnutrition	Percent	2021	0.07041855	0.08968808	0.05212602
Deaths	Male	All ages	Communicable, maternal, neonatal, and nutritional diseases	High body-mass index	Percent	2021	0.01243767	0.02929325	0.0029172
Deaths	Female	All ages	Communicable, maternal, neonatal, and nutritional diseases	High body-mass index	Percent	2021	0.02086689	0.04273568	0.00569898
Deaths	Male	All ages	Injuries	Alcohol use	Percent	2021	0.00318021	0.00559259	0.00085122
Deaths	Female	All ages	Injuries	Alcohol use	Percent	2021	6.71E-05	0.0001526	6.47E-06
Deaths	Male	All ages	Communicable, maternal, neonatal, and nutritional diseases	High fasting plasma glucose	Percent	2021	0.00981642	0.01805827	0.00561987
Deaths	Female	All ages	Communicable, maternal, neonatal, and nutritional diseases	High fasting plasma glucose	Percent	2021	0.01231936	0.01828863	0.00827334
Deaths	Male	All ages	Non-communicable diseases	Childhood maltreatment	Percent	2021	1.38E-06	3.55E-06	1.98E-07
Deaths	Female	All ages	Non-communicable diseases	Childhood maltreatment	Percent	2021	5.49E-06	1.35E-05	7.91E-07
Deaths	Male	All ages	Injuries	Tobacco	Percent	2021	0.0040511	0.0052367	0.00302829
Deaths	Female	All ages	Injuries	Tobacco	Percent	2021	0.00051006	0.00073337	0.00032351
Deaths	Male	All ages	Non-communicable diseases	High systolic blood pressure	Percent	2021	0.3337612	0.38817854	0.27377766
Deaths	Female	All ages	Non-communicable diseases	High systolic blood pressure	Percent	2021	0.25551803	0.31407048	0.20406804

Deaths	Male	All ages	Non-communicable diseases	Low physical activity	Percent	2021	0.02023779	0.03259969	0.00889449
Deaths	Female	All ages	Non-communicable diseases	Low physical activity	Percent	2021	0.03479241	0.05325203	0.0151691
Deaths	Female	All ages	Injuries	Intimate partner violence	Percent	2021	0.05397472	0.07865638	0.03387808
Deaths	Male	All ages	Injuries	Low bone mineral density	Percent	2021	0.05445254	0.06283946	0.04467364
Deaths	Female	All ages	Injuries	Low bone mineral density	Percent	2021	0.06069789	0.06983223	0.04898964
Deaths	Male	All ages	Communicable, maternal, neonatal, and nutritional diseases	Drug use	Percent	2021	0.02448484	0.03065523	0.01944826
Deaths	Female	All ages	Communicable, maternal, neonatal, and nutritional diseases	Drug use	Percent	2021	0.00832422	0.01104346	0.00618435
Deaths	Male	All ages	Injuries	Occupational risks	Percent	2021	0.04107008	0.05723431	0.02839808
Deaths	Female	All ages	Injuries	Occupational risks	Percent	2021	0.00455757	0.00660676	0.00307625
Deaths	Female	All ages	Non-communicable diseases	Unsafe sex	Percent	2021	0.00269468	0.00351701	0.00223831
Deaths	Male	All ages	Communicable, maternal, neonatal, and nutritional diseases	Dietary risks	Percent	2021	0.00198927	0.00447389	0.00052327
Deaths	Female	All ages	Communicable, maternal, neonatal, and nutritional diseases	Dietary risks	Percent	2021	0.00334287	0.00637765	0.0009422
Deaths	Male	All ages	Injuries	Suboptimal temperature	Percent	2021	0.04815738	0.10631093	-0.0250349
Deaths	Female	All ages	Injuries	Suboptimal temperature	Percent	2021	0.04256158	0.10116356	-0.0254146
Deaths	Female	All ages	Communicable, maternal, neonatal, and nutritional diseases	Intimate partner violence	Percent	2021	0.00569751	0.0097187	0.00284742
Deaths	Male	All ages	Non-communicable diseases	Suboptimal temperature	Percent	2021	0.10274812	0.13746947	0.06840794
Deaths	Female	All ages	Non-communicable diseases	Suboptimal temperature	Percent	2021	0.09469811	0.12386275	0.06273296
Deaths	Male	All ages	Communicable, maternal, neonatal, and nutritional diseases	Low physical activity	Percent	2021	0.00093814	0.00203572	0.00024315
Deaths	Female	All ages	Communicable, maternal, neonatal, and nutritional diseases	Low physical activity	Percent	2021	0.00180028	0.00361909	0.00051426
Deaths	Male	All ages	Communicable, maternal, neonatal, and nutritional diseases	Unsafe sex	Percent	2021	0.0161232	0.02046084	0.01260549
Deaths	Female	All ages	Communicable, maternal, neonatal, and nutritional diseases	Unsafe sex	Percent	2021	0.05041766	0.06146588	0.04109958
Deaths	Male	All ages	Non-communicable diseases	Impaired kidney function	Percent	2021	0.16556694	0.19391007	0.12841561
Deaths	Female	All ages	Non-communicable diseases	Impaired kidney function	Percent	2021	0.19010092	0.21357411	0.15786502
Deaths	Male	All ages	Injuries	Drug use	Percent	2021	0.00126772	0.0031656	0.00034125
Deaths	Female	All ages	Injuries	Drug use	Percent	2021	0.00214899	0.0057651	0.00060363
Deaths	Male	All ages	Non-communicable diseases	Air pollution	Percent	2021	0.20886748	0.24829916	0.17208248
Deaths	Female	All ages	Non-communicable diseases	Air pollution	Percent	2021	0.17598251	0.20534802	0.14502762

Deaths	Male	All ages	Non-communicable diseases	Other environmental risks	Percent	2021	0.05394166	0.11436246	-0.0047986
Deaths	Female	All ages	Non-communicable diseases	Other environmental risks	Percent	2021	0.03427537	0.07245538	-0.0031044
Deaths	Male	All ages	Non-communicable diseases	Tobacco	Percent	2021	0.17436427	0.21383525	0.13594741
Deaths	Female	All ages	Non-communicable diseases	Tobacco	Percent	2021	0.04859352	0.06598427	0.03139006
Deaths	Male	All ages	Communicable, maternal, neonatal, and nutritional diseases	Suboptimal temperature	Percent	2021	0.04622848	0.07724132	0.00938402
Deaths	Female	All ages	Communicable, maternal, neonatal, and nutritional diseases	Suboptimal temperature	Percent	2021	0.05743254	0.09596849	0.01279884
Deaths	Male	All ages	Non-communicable diseases	Drug use	Percent	2021	0.00683271	0.01394102	0.00147293
Deaths	Female	All ages	Non-communicable diseases	Drug use	Percent	2021	0.00101818	0.00228165	0.00018709
